# Antibiotic–Cyclodextrin Interactions: An Effective Strategy for the Encapsulation of Environmental Contaminants

**DOI:** 10.3390/molecules30224359

**Published:** 2025-11-11

**Authors:** Diana M. Galindres-Jiménez, Marta F. Matias, Isabel Paiva, Sónia I. G. Fangaia, Ana C. F. Ribeiro, Artur J. M. Valente, Miguel A. Esteso

**Affiliations:** 1 Facultad de Ciencias de la Salud, Universidad Católica de Ávila, Calle Los Canteros s/n, 05005 Ávila, Spain; dianam.galindres@ucavila.es (D.M.G.-J.); mangel.esteso@ucavila.es (M.A.E.); 2 Grupo de Investigación en Ciencias y Educación (ICE), Facultad de Ingeniería, Universidad de América, Cra 1 #20-53, Bogotá 111711, Colombia; 3 CQC-IMS, Department of Chemistry, University of Coimbra, Rua Larga, 3004-535 Coimbra, Portugal; marta01fmatias@gmail.com (M.F.M.); sfangaia@fmed.uc.pt (S.I.G.F.); avalente@ci.uc.pt (A.J.M.V.); 4 Centre of Geography and Spatial Planning, Department of Geography and Tourism, University of Coimbra, 3004-530 Coimbra, Portugal; isabelrp@fl.uc.pt; 5 Center for Innovation and Research in Oral Sciences (CIROS), Institute of Implantology and Prosthodontics, University of Coimbra, Av. Bissaya Barreto, Blocos de Celas, 3000-075 Coimbra, Portugal; 6 Unidad Docente Química Física, Universidad de Alcalá, 28805 Alcalá de Henares, Spain

**Keywords:** cyclodextrins, density, diffusion coefficient, solutions, sulfamethoxazol, trimetoprin, viscosity

## Abstract

This study reports measurements of density, viscosity, and ternary mutual diffusion coefficients (*D*_11_, *D*_12_, *D*_21_, *D*_22_) for aqueous solutions containing two antibiotics—sulfamethoxazole (SMX) or trimethoprim (TMP) (component 1)—in the presence of various cyclodextrins (α–CD, β–CD, and γ–CD) (component 2) at 298.15 K. The relative viscosity data were analyzed by fitting to a second-order Jones-Dole equation via a least-squares regression to obtain the viscosity *B* coefficients. Apparent molar volumes (*V_ϕ_*) were derived from the measured densities (*ρ*) for SMX and TMP in aqueous media. Furthermore, partial molar volumes of transfer at infinite dilution, Δ*V_ϕ_*^0^, were evaluated to elucidate solute–solvent interactions within the ternary systems investigated. Nonzero Δ*V_ϕ_*^0^ values, positive viscosity *B* coefficients, and negative cross-diffusion coefficients (*D*_12_ and *D*_21_), evidencing significant coupled diffusion, collectively indicate strong interactions between the antibiotics and cyclodextrins, consistent with host–guest complex formation.

## 1. Introduction

In recent decades, accelerated population growth and urban expansion have imposed considerable pressure on aquatic ecosystems, predominantly as a consequence of both direct and indirect pollutant discharges arising from diverse anthropogenic activities. The swift urbanization process, coupled with the release of industrial, domestic, and agricultural effluents, alongside the conveyance of contaminants through stormwater runoff, has precipitated notable degradation in water resource quality. This context highlights the imperative for sustainable urban water cycle management, grounded in a comprehensive framework that synthesizes scientific, technical, ecological, and socioeconomic knowledge [1,2]. Among the pollutants that have gathered increasing attention in recent years are emerging organic pollutants (EOPs), also referred to as emerging contaminants—substances that, although not yet regulated, are increasingly detected in aquatic environments and pose considerable risks to both environmental integrity and public health. These contaminants encompass a broad spectrum of compounds, including pharmaceuticals, hormones, industrial additives, microplastics, and nanomaterials. Although typically detected at low concentrations, they raise significant concerns due to their inherent toxicity and potential for bioaccumulation within aquatic organisms [3,4]. Although the pharmaceutical industry invests substantially in research to ensure drug safety and efficacy, the environmental impacts of these compounds have received comparatively limited attention. The release of pharmaceuticals and their metabolites into aquatic and terrestrial ecosystems can result in contamination of soil and water resources, adversely affecting biodiversity and posing potential risks to both human and animal health. The complex interactions between pharmaceuticals and environmental matrices, coupled with the scarcity of comprehensive data on their environmental occurrence and fate, underscore the urgent need for further research in this critical area [5]. Among the various classes of pharmaceuticals detected in the environment, antibiotics warrant particular attention due to their role in the emergence and proliferation of bacterial resistance, which constitutes one of the most pressing public health challenges globally [6,7]. Widely utilized in both human and veterinary medicine, these compounds can disrupt natural microbiological processes even at trace concentrations, thereby facilitating the development and dissemination of antibiotic-resistant bacteria [8]. The rising concentrations of antibiotics in aquatic environments reflect not only patterns of consumption but also the inherent limitations of current wastewater treatment technologies [9]. Trimethoprim (TMP), in turn, acts as an inhibitor of the bacterial enzyme dihydrofolate reductase (DHFR), thereby blocking a subsequent step in the folic acid biosynthetic pathway. The combination of sulfamethoxazole (SMX) and TMP produces a synergistic effect by sequentially inhibiting two critical enzymatic steps in folic acid synthesis, which impedes the development of bacterial resistance [10]. This combination is extensively employed in the treatment of urinary, respiratory, and gastrointestinal tract infections in both hospital and outpatient settings. Typically, these drugs are administered orally in pharmaceutical formulations with concentrations ranging from 250 to 500 mg L^−1^, exhibiting adsorption rates of approximately 70–90%. The primary elimination route is renal excretion, with about 20% of the administered dose excreted unmetabolized following hepatic metabolism. Despite advances in contaminant removal technologies, significant gaps remain in addressing emerging pollutants, including pharmaceutical residues and microplastics. The development of host molecules—such as cyclodextrins, crown ethers, and calixarenes—has become a focal point in this field, owing to their capacity to selectively recognize and interact with target molecules [11,12].

**Figure 1:**
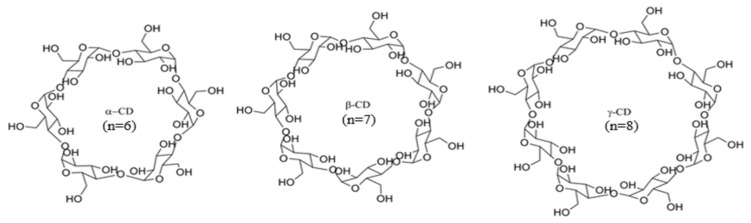
Alpha-, beta- and gamma-cyclodextrins (α–CD, β–CD, and γ–CD).

Cyclodextrins (CDs) are cyclic oligosaccharides composed of repeating D-glucose units linked by α– (1→4) glycosidic bonds, forming a toroidal structure characterized by a relatively nonpolar inner cavity and a hydrophilic exterior surface due to the orientation of hydroxyl groups. This unique architecture enables the formation of inclusion complexes through the insertion of guest molecules—typically nonpolar—into the CD cavity, stabilized by hydrophobic interactions, but also allows the interaction of guest molecules with the hydroxyl groups located at both rims of the CD by, e.g., hydrogen bonding [13,14]. The most common natural CDs are α–CD, β–CD, and γ–CD, which differ in the number of glucose units (6, 7, and 8 units, respectively) resulting in distinct cavity diameters (Figure 1).

This variation influences their inclusion capacity and selectivity towards specific guest molecules. For instance, β–CD is extensively utilized due to its commercial availability and lower cost, despite exhibiting lower aqueous solubility compared to α– and γ–CDs. These properties are extensively exploited in the pharmaceutical industry to enhance the solubility, stability, and sustained release of hydrophobic drugs, while also mitigating adverse effects. CDs have garnered increasing attention as polymeric matrices for the removal and sensing of emerging contaminants, including pharmaceuticals and organic pollutants. Their capacity to form stable inclusion complexes facilitates the extraction, sequestration, and potential degradation of such compounds [15].

To the best of our knowledge, no data are currently available regarding the ternary mutual diffusion coefficients of the pharmaceutical compounds SMX and TMP in aqueous solutions containing CDs. This study aims to fill this gap by presenting experimental diffusion coefficient data obtained via the Taylor dispersion method at 298.15 K for aqueous ternary systems—SMX/CDs (α–, β–, and γ–CD) and TMP/CDs (α–, β–, and γ–CD)—at different carrier concentrations. These measurements are complemented by the quantification of other thermodynamic and transport parameters, such as density and viscosity, and by spectroscopy studies. All together data are expected to provide valuable insights into the molecular interactions between cyclodextrin carriers and the drugs. The findings contribute to a deeper understanding of complexation mechanisms and may support the development of novel formulations in the field of drug delivery.

## 2. Results and Discussion

### 2.1. Binary and Ternary Mutual Diffusion Coefficients of Aqueous Systems Containing SMX and TMP

Table 1 shows the mean values of binary diffusion coefficients, *D*, of SMX and TMP in aqueous solutions at infinitesimal and 0.0010 mol kg^−1^ and respective experimental standard deviations of the mean values of diffusion coefficients, *S*_D_, at 298.15 K. Table 2 and Table 3 present the ternary mutual diffusion coefficients for six aqueous systems (SMX or TMP, component 1) plus CDs at different compositions, *m*_1_ and *m*_2_, at 298.15 K.

The analysis of diffusion coefficients in ternary systems is inherently more complex than in binary systems, as it takes into account the influence of the concentration gradient of one solute on the transport of the other. This is particularly relevant in the case of pharmaceutical compounds (SMX and TMP) and CDs in aqueous solutions (Table 2 and Table 3).

Mutual diffusion coefficients for ternary solutions {drug (1) + CD (2) + water} are described by the coupled Fick’s Equations (1) and (2):

*J*_1_ = −*D*_11_∇*C*_1_ − *D*_12_∇*C*_2_ (1)

*J*_2_ = −*D*_21_∇*C*_1_ − *D*_22_∇*C*_2_ (2)

where *J*_1_ and *J*_2_ represent the molar fluxes of solute (1) and solute (2) driven by the concentration gradients ∇*C*_1_ and ∇*C*_2_ in solution, respectively. *D*_11_ and *D*_22_ are the so-called main diffusion coefficients, which allow assessing the molar fluxes of solute (1) and solute (2) driven by their own concentration gradients. *D*_12_ and *D*_21_ are the cross-diffusion coefficients, which provide information on the coupled flux of each solute, 1 or 2, driven by a concentration gradient of the other solute, 2 or 1, respectively. A positive *D_ab_* (*a* ≠ *b*) cross-diffusion coefficient indicates coupled co-current transport of solute *a* from regions of higher to lower concentration of solute *b*; and a negative *D*_ab_ (*a* ≠ *b*) coefficient shows coupled counter-current transport of solute *a* from regions of lower to higher solute *b* concentration. When comparing the diffusion coefficients of each drug and the cyclodextrins (Table 1) in water with the main diffusion coefficients of the ternary systems (*D*_11_ and *D*_22_) (Table 2 and Table 3), significant deviations are observed, ranging from approximately 1% to 45%. These discrepancies can be attributed to the interactions between the two solutes present (i.e., SMX and CDs, and TMP and CDs). In particular, the values of *D*_22_ associated with CDs in ternary systems are substantially lower than the binary diffusion coefficients of the corresponding CDs, indicating a significant reduction in the mobility of CD in the presence of the drug. Such effect can be rationalized as due to supramolecular complexes or by modifications in the solvent structure or in the solvation shell of the CD in the presence of the drug.

Similarly, the values of *D*_11_ obtained for the drugs also differ from their respective binary diffusion coefficients, further supporting the hypothesis of significant intermolecular interactions in the studied systems.

The observed decrease, although not pronounced, suggests that the solute species maintain non-negligible interactions with the solvent. Additionally, there is a trend of increasing *D*_11_ with the molar fraction of component 1 (*X*_1_), while *D*_22_ tends to decrease with the same increase, though minor fluctuations in the experimental values may occur. This latter behavior indicates that the increase in the concentration of the solute (CDs) in solution is associated with greater resistance to its mobility, reflecting a diffusion-limiting effect due to more intense intermolecular interactions.

When the molar fraction of drugs (*X*_1_) approaches zero, the diffusion coefficient *D*_11_ is referred to as the “tracer” diffusion coefficient of the drugs in aqueous solutions of CDs. Thus, *D*_22_ represents the diffusion coefficient of CD in solutions of 0.001 mol·dm^−3^, and *D*_12_ is ca. zero once no drugs are in solution. However, in solutions of finite concentration (*X*_1_ > 0), generally, non-negligible negative values of *D*_12_ are observed (reaching a maximum at *X*_1_ = 1), confirming that the diffusion of CDs in these solutions creates a counteracting transport of drugs.

The presence of inclusion complexes between these drugs and CDs acting as carrier vehicles may explain the observation of negative values. Specifically, in regions where the concentration of SMX and TMP is higher, a more pronounced decrease in these “free” species will occur due to the formation of these complexes. To compensate that, a counter current flow of the drugs is observed, from regions of lower concentration to those of higher concentration of CDs. This phenomenon is more evident in systems containing TMP, suggesting a higher interaction with CD. TMP shows the strongest interaction with CDs, with the most negative value of *D*_12_ obtained in solutions containing γ–CD (*D*_12_ ≈ −0.571 × 10^−9^ m^2^ s^−1^ for *X*_1_ = 1). In the case of SMX, the most negative value for this parameter was recorded in solutions of β–CD (*D*_12_ ≈ −0.190 × 10^−9^ m^2^ s^−1^ for *X*_1_ = 1).

Regarding negative values of *D*_21_, although they are almost zero within the error limits of this method, they also indicate coupled counter current transport of CDs from regions of lower to higher drug concentration, reaching maximum values at *X*_1_ = 0. At the other limit (*X*_1_ = 1), the diffusion coefficients *D*_11_ are close to the drug diffusion coefficient in binary systems for the same concentration (*D* = 0.666 × 10^−9^ m^2^ s^−1^). *D*_22_ represents the “tracer” diffusion coefficient of CDs in aqueous solutions containing these drugs. *D*_21_ = 0, as the concentration gradient of the drugs cannot be responsible for the flux of CDs in their absence.

By calculating (*D*_12_/*D*_22_) and (*D*_21_/*D*_11_), the number of moles of the drug (SMX or TMP) transported per mole of CD, and the number of moles of CD transported per mole of drug, respectively, can be estimated. These values for each system are presented in Table 2 and Table 3.

A preliminary analysis reveals that the values of *D*_12_/*D*_22_ are consistently higher than those of *D*_21_/*D*_11_. This could be due to the size and structure of the drug molecules, which have a much lower molecular mass than CDs.

For a deeper understanding of the results obtained, a model developed by Paduano et al. [16] for systems involving the formation of inclusion complexes was applied.

Assuming the presence of supramolecular structures with a 1:1 stoichiometry between the free CDs and SMX (or TMP) in dilute solutions, it is possible to estimate values for the mutual diffusion coefficients and compare them with the experimentally obtained values, presented in Table 2 and Table 3.

Considering the equilibrium described by Equation (3) and the corresponding equilibrium constant, *K*, in Equation (4):

(3) $${\mathrm{Drug}}_{\left(\mathrm{aq}\right)}+\;{\mathrm{CD}}_{\left(\mathrm{aq}\right)}\underset{\to }{\leftarrow }\mathrm{Drug}\;-\;\mathrm{CD}\;(\mathrm{aq})$$

(4) $$K=\frac{C_{(\mathrm{Drug}-\mathrm{CD})}}{C_{\mathrm{Drug}}.\;C_{\mathrm{CD}}}$$

The diffusion coefficients of the drugs-CD complexes are estimated using the Stokes-Einstein approximation (Equation (5)) and using the limiting diffusion coefficients of free species SMX and TMP, and CDs in water (Table 4). According to this approach, the diffusion coefficient of a drug–CD complex in solution (*D*s) is inversely proportional to its hydrodynamic radius and, consequently, to the cube root of its molecular volume, as described by Equation (5).

*D*_s_ = (*D*_SMX (or TMP)_^−3^ + *D*_CDs_^−3^) ^−1/3^ (5)

*D*_SMX (or TMP)_ and *D*_CDs_ represent the limiting diffusion coefficients of SMX, TMP and CDs.

**Figure 2:**
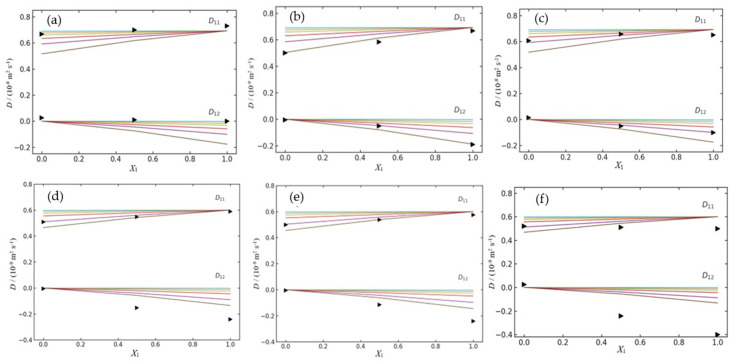
Ternary mutual diffusion coefficients, *D*_11_ and *D*_12_, as a function of the solute fraction of antibiotic, X_1_, at 298.15 K. (**a**) SMX (component 1) + α–CD (component 2); (**b**) SMX (component 1) + β–CD (component 2); (**c**) SMX (component 1) + **γ**–CD (component 2); (**d**) TMP (component 1) + α–CD (component 2); (**e**) TMP (component 1) + β–CD (component 2). (**f**) TMP (component 1) + **γ**–CD (component 2). Experimental values (►) and theoretical values calculated for different values of association constants, *K* (M^−1^): 10 (▬), 50 (▬), 100 (▬), 200 (▬), 500 (▬) and 1000 (▬).

As an illustrative example, Figure 2 present the variation of the ternary diffusion coefficients, *D*_11_ and *D*_12_, as a function of the drug molar fraction (*X*_1_) for the two ternary systems (SMX + CDs and TMP + CDs). These values are predicted by the applied model using different equilibrium constants, *K* (i.e., *K* = 10, 50, 100, 200, 500, and 1000 M^−1^), alongside the corresponding experimental data obtained within this concentration range.

The comparison between the predicted results and our data obtained by Taylor diffusion measurements allow us to draw a coherent picture of the affinity between the two drugs studied (SMX and TMP) and the three natural cyclodextrins (α–, β–, and γ–CD). For example, from the analysis of the mutual diffusion coefficients (*D*_ij_) for ternary system SMX + CDs (Table 2 and Figure 2a–c), it can be seen that the theoretical curve calculated for an association constant of *K* = 1000 M^−1^, relative to the system SMX + β–CD, is the one that most closely matches the experimental values. The simulated curves for lower *K* values (such as 10, 50, 100, or 500) clearly underestimate the *D*_11_ values, demonstrating that they do not adequately reflect the observed behavior. Concerning the dependence of *D*_12_ on the composition, both the experimental and predicted values demonstrate a slight negative trend, consistently remaining just below zero throughout the full range of mole fractions. Relative to the other ternary systems (Figure 2a,c), the association constants that yield the best fitting curves are lower. (i.e., *K* = 10 M^−1^ and 100 M^−1^ for α–CD and γ–CD, respectively).

A pronounced increase in the association constant was observed as one passes from α–CD to β–CD, followed by a moderate decrease with γ–CD: the coefficients *D*_11_ and *D*_12_ are reasonably well described when it uses *K* ≈ 10 M^−1^ for α–CD, by 500 < *K* ≤ 10^3^ M^−1^ for β–CD, and by *K* ≈ 10^2^ M^−1^ for γ–CD. This trend confirms that the cavity of β–CD (internal diameter of 6.0–6.5 Å) has dimensions more suitable for the partial inclusion of the aromatic structure of SMX, favoring the formation of a stable 1:1 complex [20]. The smaller cavity of α–CD allows only a superficial fit, whereas the enlarged cavity of γ–CD (7.5–8.3 Å) tends to self-aggregate in aqueous solution and form self-organized nanoparticles, which may affect the efficiency of inclusion [21,22].

Support for this complex formation between SMX and β–CD, and these *K* values comes from our spectroscopic data (Section 2.4, *K* = 982 M^−1^) and, also, from other studies such as spectrofluorimetric measurements (*K* = 414 M^−1^) [23,24,25].

In the case of TMP, the simultaneous fitting of *D*_11_ and *D*_12_ suggests *K* ≈ 500–1000 M^−1^ in the system with α–CD, *K* ≈ 1000 M^−1^ with β–CD, and *K* > 1000 M^−1^ with γ–CD. The *D*_12_ profile in the systems with β–CD and especially γ–CD deviates from the theoretical curves, becoming more negative than any calculated value. This behavior indicates that, in addition to the 1:1 complex, competing phenomena—such as intermolecular aggregation of TMP or the formation of multiple complexes—may be occurring, affecting the cross-diffusion coefficients and not accounted for by the adopted model.

In comparative terms, the following conclusions can be drawn: (i) SMX exhibits the highest affinity among all studied systems, with a particularly strong interaction with β–CD; (ii) TMP shows moderate interaction with both α– and β–CD, while its affinity for γ–CD tends to be higher, exceeding 1000 M^−1^; (iii) the average affinity order is β–CD > γ–CD > α–CD for SMX, and γ–CD > α–CD ≈ β–CD for TMP (Table 4).

It is important to note that this model has certain limitations, as it assumes the exclusive formation of 1:1 complex and a rapid chemical equilibrium between free and complexed species. It does not account for cooperative interactions or the formation of multiple aggregates. Therefore, the association constant obtained should be interpreted within these constraints.

### 2.2. Density and Volume Data

The density value for eight systems was the mean of at least ten sets of measurements. These values were reproducible within ±0.001% uncertainty (Tables S1–S4). Table 5 show limiting partial molar volumes, $V_{\phi }^{0}$ and experimental slopes, *b*_v_, for SMX in (water + CDs at different concentrations).

Partial molar volumes of transfer at infinite dilution, Δ$V_{\phi }^{0}$, from water {$V_{\phi }^{0}$_(in water)_} [26,27] to aqueous CDs solutions {$V_{\phi }^{0}$ _(in aqueous CDs solutions)_} (Table S2) have been calculated via Equation (6). The resulting values are listed in Table 6.

(6) $$\Delta V_{\phi }^{0}={V_{\phi }^{0}\;}_{\left(\mathrm{in}\;\mathrm{aqueous}\;\mathrm{CDs}\;\mathrm{solutions}\right)}-{V_{\phi }^{0}\;}_{\left(\mathrm{in}\;\mathrm{water}\right)}$$

These values of the transfer volumes only provide information about the interactions between the solute molecules and those of the solvent (solute-solvent interaction).

Table 7 and Table 8 show the limiting partial molar volumes, $V_{\phi }^{0},$ and experimental slopes, *b*_v_, for TMP in (water + CDs at different concentrations, and the respective transfer partial molar volumes, Δ$V_{\phi }^{0}$ for TMP in these aqueous CDs.

Analysis of the results in Table 6 and Table 8 shows that the presence of CDs in the medium leads to a decrease in the apparent molar volume of both SMX and TMP, resulting in negative values for the partial molar volumes of transfer $∆V_{\phi }^{0}$. In contrast, for TMP in the presence of 0.5 mM CDs, the $∆V_{\phi }^{0}$ values are positive.

The changes in $∆V_{\phi }^{0}$ observed when transferring the solutes (SMX and TMP) from pure water to a mixed solvent system (CDs + water) can be interpreted using the Friedman and Krishnan model [28]. According to this model, hydrophilic–hydrophobic and hydrophobic–hydrophobic interactions contribute negatively to the $∆V_{\phi }^{0}$ values, while ionic–hydrophilic and hydrophilic–hydrophilic interactions contribute positively.

Thus, based on our data, there is clear evidence supporting the formation of SMX–CDs inclusion complexes, likely driven by predominant hydrophilic–hydrophobic or hydrophobic–hydrophobic interactions within the system. Regarding the thermodynamic behavior of SMX, this effect—reflected in the negative values of $∆V_{\phi }^{0}$—is more pronounced in the presence of γ–CD, suggesting stronger interactions and a more stable complex formation with this specific CD.

### 2.3. Viscosity Data

Table S5 summarizes the mean values and corresponding standard deviations of viscosity measurements in aqueous solutions of SMX and TMP, both in the absence and presence of CDs. Although these drugs are non-electrolytes, considering the possibility of instantaneous dipoles in aqueous systems containing SMX and TMP, the influence of concentration on solution viscosity was analyzed using an equation analogous to the Jones–Dole equation (Equation (7)) [29], which is typically applied to electrolytes.

(7) $$\eta_{r}=\frac{\eta_{sol}}{\eta_{H2O}}=1+Am^{1/2}+Bm$$

In Equation (7), *η*_H2O_ and *η*_sol_ represent the viscosities of water and of the solution, respectively. The coefficients *A* and *B* are constants that depend on the solute, solvent, temperature, and pressure. The coefficient *A*, which is related to long-range intermolecular forces, is typically very small and can often be considered negligible.

The *B* coefficient is related to solute–solvent interactions in the solution. A positive viscosity coefficient *B* indicates that the solute has the ability to organize the solvent structure (structure-making), whereas a negative *B* value suggests that the solute disrupts the water structure (structure-breaking).

By fitting the experimental viscosity data (Table S5) to Equation (7) using the least-squares method, the values of coefficients *A* and *B*, along with the determination coefficients (*R*^2^) of the fit, were obtained (Table 9).

Analysis of Table 9 indicates that the *B* coefficient values for the aqueous systems are positive, suggesting that both SMX and TMP are hydrated in solution. In the presence of β–CD, the *B* coefficient increases further, reflecting enhanced solute–solvent interactions. This behavior implies that β–CD acts as a structure-making agent under these conditions. These findings are consistent with the diffusion data, which also support the formation of inclusion complexes between SMX (or TMP) and β–CD in the ternary systems.

### 2.4. Interaction Between β–CD and SMX Studied by UV-Vis Spectroscopy

A calibration curve for SMX was prepared based on absorbance measurements at a wavelength of 264 nm, yielding a linear fit up to an SMX concentration of 5 mmol·dm^−3^, with a coefficient of determination (*R*^2^) of 0.9998 and a molar absorption coefficient of 183.44 L·mol^−1^·cm^−1^. The statistical parameters of the calibration curve are summarized in Table 10, where *R*^2^ represents the coefficient of determination, LOD the limit of detection (mmol·L^−1^), and LOQ the limit of quantification (mmol·dm^−3^).

**Figure 3:**
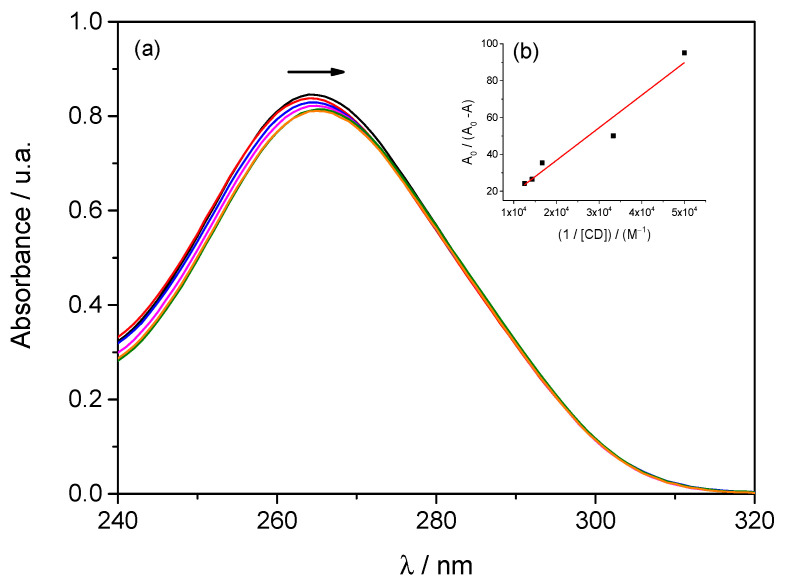
(**a**) UV–Vis absorption spectra of SMX (0.05 mmol·dm^−3^) in the presence of different concentrations of β–CD: 0.00 (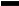); 0.02 (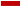); 0.03 (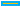); 0.06 (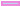); 0.07 (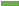); 0.08 (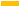) mmol·dm^−3^; (**b**) Variation of absorbance as a function of the inverse of CD concentration.

The interaction between SMX and β–CD was also evaluated using UV–Vis spectroscopy. Figure 3 shows the absorption spectra of aqueous SMX solutions in the presence of β–CD. In this case, absorption spectra were recorded for SMX at a fixed concentration of 0.05 mmol·dm^−3^, both in the absence and presence of β–CD, with β–CD concentrations ranging from 0.02 to 0.08 mmol·dm^−3^.

The analysis of the UV-Vis spectra (Figure 3) reveals that the gradual addition of β–CD to SMX causes a continuous decrease in the maximum absorbance (hypochromic effect) and a slight shift of the peak to lower wavelengths (hypochromic shift). These phenomena suggest the formation of SMX–β–CD inclusion complexes, in which SMX is partially encapsulated by the hydrophobic cavity of the CD, modifying the local environment of the chromophore.

The association constant between SMX and β–CD was determined using the Benesi–Hildebrand method [30], based on absorbance values obtained for a 0.05 mmol·dm^−3^ SMX solution in the presence of β–CD at varying concentrations (0.02–0.08 mmol·dm^−3^). Since the formation of the inclusion complex led to a decrease in absorbance (*A* < *A*_0_), a normalized representation of the data was used (Equation (8)).

(8) $$\frac{A_{0}}{A-A_{0}}=\frac{A_{0}}{K\;∆A_{max}\;\left[CD\right]}+\frac{A_{0}}{∆A_{máx}}$$

$A_{0}\;$ is the absorbance of SMX without CD, A is the absorbance in the presence of CD, *K* is the association constant, $∆A_{max}$ is the maximum change in absorbance, and [CD] is the concentration of β–CD.

Figure 3 also illustrates the analysis conducted using the graphical representation of A_0_/(A − A_0_) plotted against 1/[CD]. The linearity observed in the Benesi-Hildebrand plot supports the existence of a 1:1 stoichiometry between SMX and β–CD.

The fitting of Equation (8) to the experimental data resulted in a slope of 0.00174 mol^−1^ dm^3^ and a y-intercept of 1.71, with a coefficient of determination, *R*^2^, equal to 0.940.

Based on these fitting parameters, the association constant (*K*) between SMX and β–CD was estimated to be 982 ± 300 M^−1^ for the SMX:β–CD system. Although the deviation associated with the calculated constant is relatively high, this behavior can be attributed to the low molar absorptivity of SMX in the studied region, which leads to only slight changes in absorbance with increasing β–CD concentration, thereby amplifying the error in quantitative analysis. However, it should be noted that the difference between this value and those reported in the literature (*K* = 414 M^−1^) [25] is not very large, and it is close to the value obtained for diffusion data (500 ≤ *K* ≤ 1000 M^−1^).

## 3. Materials and Methods

### 3.1. Materials

Table 11 describes all the reagents used in the present work. All chemicals were used without further purification. The weighing was performed using a Radwag AS 220C2 balance (Radwag, Radom, Poland), with an accuracy of ±0.0001 g.

### 3.2. Experimental Techniques

#### 3.2.1. Mutual Diffusion Coefficients by Taylor Dispersion Technique

The Taylor dispersion technique enables the determination of diffusion coefficients in multicomponent systems and is founded on the seminal work of G. I. Taylor in the 1950s. This method has since been extensively described, developed, and validated in the scientific literature [31]. A summary of the most relevant aspects of the Taylor dispersion technique will be presented in the following section. As with all chromatographic-based methods, the technique involves the generation of a dispersion profile by injecting a fixed volume of solution—specifically 0.063 mL—at the beginning of the experiment into a laminar carrier solution of slightly different composition into Teflon tube with a length of 3048.0 ± 0.1 cm and an internal diameter of 0.06440 ± 0.00006 cm.

All equipment is thermostated at a constant temperature of 298.15 ± 0.01 K. The dispersion profiles are recorded using a differential refractometer (Waters model 2410, Milford, MA, USA). This instrument monitors changes in refractive index by measuring the electric potential as a function of time, *V*(*t*), with the signal acquired through a digital voltmeter (Agilent 34401A, Santa Clara, CA, USA) connected to the outlet of the dispersion tube. Binary and ternary diffusion coefficients were determined by fitting Equations (9) and (10) to the experimental dispersion profiles, as described in the literature [31,32,33],

(9) $$V\left(t\right)=V_{0}+V_{1}t+{V_{max}(t_{R}/t)}^{1/2}\;exp[-12D{\left(t-t_{R}\right)}^{2}/{r\;}^{2}t]$$

(10) $$V\left(t\right)=V_{0}+V_{1}t+V_{max}{\left(t_{R}/t\right)}^{1/2}\left[W_{1}exp\left(-\frac{12D_{1}{\left(t-t_{R}\right)}^{2}}{r^{2}t}\right)+(1-W_{1})exp\left(-\frac{12D_{2}{\left(t-t_{R}\right)}^{2}}{r^{2}t}\right)\right]$$

where *r*, *t_R_*, *V*_max_, *V*_0_ and *V*_1_ represent the radius of the dispersion tube, mean sample retention time, peak height, baseline voltage and baseline slope, respectively,. and *W*_1_ and (1 − *W*_1_) are normalized pre–exponential factors. In Equation (10), *D*_1_ and *D*_2_ represents the eigenvalues of the matrix of the ternary *D*_ik_ coefficients.

#### 3.2.2. Density Measurements

The density of solutions was determined using an Anton Paar (Graz, Austria) DMA 5000M densimeter, which offers a precision of 1 × 10^−6^ g·cm^−3^ and an accuracy of 5 × 10^−6^ g·cm^−3^ within the operational ranges of 0 to 90 °C and 0 to 1.0 MPa. The instrument is equipped with a Peltier temperature control system, allowing for precise thermal regulation of the samples within ±0.005 °C. All measurements were performed at 298.15 K. The estimated measurement uncertainty for the density values was 0.001%.

#### 3.2.3. Viscosity

Relative viscosity measurements of the prepared solutions were carried out using an Ubbelohde-type suspended-level viscometer. To ensure thermal equilibrium and minimize temperature fluctuations, the viscometer containing the sample was immersed in a thermostated water bath for approximately 45 min prior to measurement. The instrument was calibrated by using Milli-Q water over the temperature range of 298.15 to 313.15 K. For each solution, the efflux time was measured in triplicate using a digital stopwatch with a precision of ±0.01 s, and the average of the recorded values was used in the viscosity calculations. The estimated measurement uncertainty for viscosity was 0.01%.

#### 3.2.4. Ultraviolet–Visible (UV–Vis) Spectroscopy

UV-Vis absorption spectra of SMX at a fixed concentration of 0.05 mmol·dm^−3^ were recorded in the absence and presence of β–CD, with the concentration of β–CD varying from 0.02 to 0.08 mmol·dm^−3^. UV–Vis spectra were recorded using a Shimadzu (Tokyo, Japan) UV–2600i spectrophotometer in the wavelength range of 200 to 800 nm, employing a quartz cuvette with a 1 cm optical path length. All measurements were performed at 298.15 K.

In the case of TMP, no reliable UV-Vis data was obtained due to its low solubility in aqueous media and low molar absorption coefficient compared to some other compounds.

## 4. Conclusions

The effect of α–CD, β–CD, and γ–CD on the diffusion behavior of aqueous SMX and TMP solutions was systematically investigated. Intermolecular diffusion studies confirmed significant interactions between these antibiotics and the cyclodextrins, as evidenced by the negative values obtained for the cross-diffusion coefficients *D*_12_ and *D*_21_. These negative values indicate coupled fluxes resulting from supramolecular complex formation.

The equilibrium binding constants (*K*) for these interactions were estimated as follows: for SMX:α–CD and SMX:γ–CD, approximately 10 M^−1^ and 100 M^−1^, respectively; and for SMX:β–CD, within the range of 500–1000 M^−1^. Interactions between TMP and all three cyclodextrins (α–CD, β–CD, and γ–CD) were also observed. In this case, the estimated *K* value for TMP:α–CD falls within the 500–1000 M^−1^ range, while for TMP:β–CD and TMP:γ–CD, the binding constants are *K* ≥ 1000 M^−1^.

These results were further supported by complementary viscosity, density, and UV-Vis spectroscopy measurements, all of which consistently confirmed the formation of host–guest complexes. For instance, UV-Vis spectral analysis revealed a possible association between SMX and β–CD, with an estimated association constant (*K*) of 982 M^−1^.

Importantly, these interactions have environmental relevance: CDs offer a dual advantage by combining selective retention of pharmaceutical contaminants with the potential for regeneration of the encapsulating agents. This makes them promising candidates for application in water treatment technologies aimed at mitigating antibiotic pollution.

## Figures and Tables

**Table 1:** Binary diffusion coefficients, *D*, of SMX and TMP in aqueous solutions at infinitesimal and 0.001 mol kg^−1^ and the respective experimental standard deviations, *S*_D_, at 298.15 K.

*m*_(SMX)_ /(mol kg^−1^)	(*D* ± S_D_) /(10^−9^ m^2^·s^−1^)	*m*_(TMP)_ /(mol kg^−1^)	(*D* ± S_D_) /(10^−9^ m^2^·s^−1^)
0.0000	0.700 ± 0.020 ^a^	0.0000	0.600 ± 0.020 ^b^
0.0010	0.670 ± 0014	0.0010	0.580 ± 0.013

^a^ Estimate of the diffusion coefficient at infinitesimal concentration obtained by linearly fitting the equation *D*/(10^−9^ m^2^·s^−1^) = −5.43 *m* + 0.725 to the experimental data *D* = 0.710, 0.700, 0.670 × 10^−9^ m^2^·s^−1^, obtained by injection solutions at *m* = 0.0025, 0.005 and 0.0010 mol kg^−1^ in water. ^b^ Value obtained from injection solution 0.0010 mol kg^−1^ in water. Standard uncertainties are: *u*(*m*) = 1.0 × 10^−3^ mol kg^−1^ (max); u(*T*) = 0.01 K; u(*P*) = 2.03 kPa.

**Table 2:** Ternary diffusion coefficients, *D*_11_, *D*_12_, *D*_21_ and *D*_22_, for SMX (component 1) + α, β and γ–CDs (component 2) and their respective standard deviations.

*m*_1_^a^	*m*_2_^a^	*X*_1_^b^	*D*_11_ ± *S*_D_^c^	*D*_12_ ± *S*_D_^c^	*D*_21_ ± *S*_D_^c^	*D*_22_ ± *S*_D_^c^	*D*_12_*/D*_22_ ^d^	*D*_21_*/D*_11_ ^e^
SMX (*m*_1_) + α–CD (*m*_2_)								
0.0000	0.0010	0.000	0.668 ± 0.026	0.004 ± 0.010	0.042 ± 0.010	0.394 ± 0.012	0.010	0.006
0.0005	0.0005	0.500	0.700 ± 0.020	−0.026 ± 0.015	−0.037 ± 0.030	0.379 ± 0.019	−0.069	−0.053
0.0010	0.0000	1.000	0.731 ± 0.012	−0.048 ± 0.011	−0.079 ± 0.020	0.364 ± 0.010	−0.132	−0.108
SMX (*m*_1_) + β–CD (*m*_2_)								
0.0000	0.0010	0.000	0.500 ± 0.114	−0.005 ± 0.019	0.003 ± 0.007	0.300 ± 0.017	−0.333	0.006
0.0005	0.0005	0.500	0.584 ± 0.013	−0.050 ± 0.025	0.001 ± 0.010	0.239 ± 0.016	0.367	0.005
0.0010	0.0000	1.000	0.667 ± 0.006	−0.190 ± 0.012	−0.002 ± 0.009	0.178 ± 0.009	1.067	0.003
SMX (*m*_1_) + γ–CD (*m*_2_)								
0.0000	0.0010	0.000	0.607 ± 0.020	0.015 ± 0.013	0.001 ± 0.001	0.374 ± 0.004	0.040	0.0016
0.0005	0.0005	0.500	0.658 ± 0.010	−0.050 ± 0.010	0.001 ± 0.001	0.386 ± 0.006	−0.130	0.0152
0.0010	0.0000	1.000	0.650 ± 0.012	−0.100 ± 0.011	0.019 ± 0.020	0.391 ± 0.007	−0.256	0.029

^a^ *m*_1_ and *m*_2_ in mol kg^−1^. ^b^ *X*_1_ = *m*_1_/(*m*_1_ + *m*_2_) for ternary system. ^c^ (*D* ± *S*_D_)/(10^−9^ m^2^ s^−1^) represents the mean diffusion coefficients of 4 to 6 replicate measurements, and the corresponding standard deviation of the mean. ^d^ *D*_12_/*D*_22_ is the number of moles of SMX transported per mole of CD. ^e^ *D*_21_/*D*_11_ is the number of moles of CD transported per mole of SMX. Standard uncertainties are: *u*_r_ (*m*) = 1.0 × 10^−3^ (max); *u*(*T*) = 0.01 K; *u*(*P*) = 2.03 kPa.

**Table 3:** Ternary diffusion coefficients, *D*_11_, *D*_12_, *D*_21_ and *D*_22_, for TMP (component 1) + α, β and γ–CDs (component 2) and their respective standard deviations.

*m*_1_^a^	*m*_2_^a^	*X*_1_^b^	*D*_11_ ± *S*_D_^c^	*D*_12_ ± *S*_D_^c^	*D*_21_ ± *S*_D_^c^	*D*_22_ ± *S*_D_^c^	*D*_12_*/D*_22_^d^	*D*_21_*/D*_11_^e^
TMP (*m*_1_) + α–CD (*m*_2_)								
0.0000	0.0010	0.000	0.510 ± 0.019	−0.004 ± 0.010	−0.050 ± 0.011	0.330 ± 0.009	−0.012	−0.098
0.0005	0.0005	0.500	0.548 ± 0.012	−0.152 ± 0.020	−0.030 ± 0.014	0.346 ± 0.010	−0.439	−0.058
0.0010	0.0000	1.000	0.589 ± 0.008	−0.240 ± 0.022	−0.009 ± 0.009	0.340 ± 0.008	−0.706	−0.015
TMP (*m*_1_) + β–CD (*m*_2_)								
0.0000	0.0010	0.000	0.500 ± 0.011	−0.004 ± 0.010	−0.150 ± 0.011	0.360 ± 0.007	−0.011	−0.300
0.0005	0.0005	0.500	0.538 ± 0.020	−0.138 ± 0.022	−0.074 ± 0.036	0.332 ± 0.010	−0.416	−0.138
0.0010	0.0000	1.000	0.576 ± 0.010	−0.271 ± 0.030	0.001 ± 0.037	0.305 ± 0.004	−0.889	0.002
TMP (*m*_1_) + γ–CD (*m*_2_)								
0.0000	0.0010	0.000	0.520 ± 0.010	0.026 ± 0.020	0.039 ± 0.030	0.320 ± 0.001	0.081	0.075
0.0005	0.0005	0.500	0.510 ± 0.026	−0.272 ± 0.030	−0.016 ± 0.030	0.309 ± 0.010	−0.880	0.031
0.0010	0.0000	1.000	0.500 ± 0.020	−0.571 ± 0.060	−0.004 ± 0.009	0.299 ± 0.014	−1.910	−0.008

^a^ *m*_1_ and *m*_2_ in mol kg^−1^. ^b^ *X*_1_ = *m*_1/_(*m*_1_ + *m*_2_) for ternary system. ^c^ (*D* ± *S*_D_)/(10^−9^ m^2^ s^−1^) represents the mean diffusion coefficients of 4 to 6 replicate measurements, and the corresponding standard deviation of the mean. ^d^ *D*_12_/*D*_22_ is the number of moles of TMP transported per mole of CD. ^e^ *D*_21_/*D*_11_ is the number of moles of CD transported per mole of TMP. Standard uncertainties are: *u*_r_ (*m*) = 1.0 × 10^−3^ (max); *u*(*T*) = 0.01 K; *u*(*P*) = 2.03 kPa.

**Table 4:** Limiting diffusion coefficients of species, *D*_s_, at 298.15 K.

Species	*D*_s/_(10^−9^ m^2^ s^−1^)
SMX	0.700 ^a^
TMP	0.600 ^a^
α–CD	0.353 ^b^
β–CD	0.326 ^c^
γ–CD	0.311 ^d^
SMX: α–CD ^b^	0.353 ^e^
SMX: β–CD	0.315 ^e^
SMX: γ–CD	0.343 ^e^
TMP: α–CD	0.319 ^e^
TMP: β–CD	0.300 ^e^
TMP: γ–CD	0.322 ^e^

^a^ Limiting diffusion coefficients of free species at 0.001 mol·dm^−3^ in water (Table 1). ^b^ Value from [17], ^c^ Value from [18], ^d^ Value from [19], ^e^ These values were estimated from Equation (5) using the limiting diffusion coefficients of the involved species.

**Table 5:** Limiting partial molar volumes, $V_{\phi }^{0}$, and experimental slopes, *b*_v_, for SMX in (water + CDs) mixed solvents at different concentrations, *m*, at *T* = 298.15 K and at pressure *P* = 101.3 kPa.

*m*(CD) /(mol kg^−1^)	$V_{\phi }^{0}/$ (cm^3^·mol^−1^) (in α–CD) ^a^	*b*_v_/ (kg·cm^3^·mol^−2^)	$V_{\phi }^{0}$/ (cm^3^·mol^−1^) (in β–CD) ^a^	*b*_v_/ (kg·cm^3^·mol^−2^)	$V_{\phi }^{0}$/ (cm^3^·mol^−1^) (in γ–CD) ^a^	*b*_v_/ (kg·cm^3^·mol^−2^)
0.00050	173.0_4_	+5080	172.0_2_	+2.0	177.0_5_	+5080
0.00100	174.5_2_	+12,188	180.0_5_	+8030	161.9_6_	+16,064

^a^ See Table S2 in Supplementary Materials. Standard uncertainties are: *u_r_*(*m*) = 1.0 × 10^−3^ (max); *u*(*V_ϕ_*) = 0.1 cm^3^·mol^−1^; *u*(*T*) = 0.01 K; *u*(*P*) = 2.03 kPa.

**Table 6:** Transfer partial molar volumes, Δ$V_{\phi }^{0}$, for SMX in aqueous CDs (α–, β–, and γ–CD) solutions at different concentrations, *m*.

*m*_(CDs)_ /(mol kg^−1^)	$\Delta V_{\phi }^{0}$/ (cm^3^·mol^−1^) (in α–CD) ^a^	$\Delta V_{\phi }^{0}$/ (cm^3^·mol^−1^) (in β–CD) ^a^	$\Delta V_{\phi }^{0}$/ (cm^3^·mol^−1^) (in γ–CD) ^a^
0.00050	−24.1_5_	−25.1_7_	−19.4_3_
0.00100	−22.6_7_	−17.1_4_	−35.2_3_

^a^ $∆V_{\phi }^{0}$ = $V_{\phi }^{0}$_(in aqueous CDs solutions)_ − $V_{\phi }^{0}$_(in water)_.${\;V}_{\phi }^{0}$ for SMX in water = 197.1_9_ cm^3^·mol^−1^ (Table S1).

**Table 7:** Limiting partial molar volumes, $V_{\phi }^{0}$, and experimental slopes, *b*_v_, for TMP in (water + CDs) mixed solvents at different concentrations, *m*, at *T* = 298.15 K and at pressure *P* = 101.3 kPa.

*m*(CD) /(mol kg^−1^)	$V_{\phi }^{0}$/ (cm^3^·mol^−1^) (in α–CD) ^a^	*b*_v_/ (kg·cm^3^·mol^−2^)	$V_{\phi }^{0}$/ (cm^3^·mol^−1^) (in β–CD) ^a^	*b*_v_/ (kg·cm^3^·mol^−2^)	$V_{\phi }^{0}$/ (cm^3^·mol^−1^) (in γ–CD) ^a^	*b*_v_/ (kg·cm^3^·mol^−2^)
0.00050	244.8_7_	+8040	232.3_0_	+11,920	221.7_4_	+21,712
0.00100	207.1_5_	+12,176	206.1_4_	+17,264	205.1_2_	+6816

^a^ See Table S4 in Supplementary Materials. Standard uncertainties are: *u_r_*(*m*) = 1.0 × 10^−3^ (max); *u*(*V_ϕ_*) = 0.1 cm^3^·mol^−1^; *u*(*T*) = 0.01 K; *u*(*P*) = 2.03 kPa.

**Table 8:** Transfer partial molar volumes, Δ$V_{\phi }^{0}$, for TMP in aqueous CDs (α–, β–, and γ–CD) solutions at different concentrations, *m.*

*m*_(CDs)_ /(mol kg^−1^)	$\Delta V_{\phi }^{0}$/ (cm^3^·mol^−1^) (in α–CD) ^a^	$\Delta V_{\phi }^{0}$/ (cm^3^·mol^−1^) (in β–CD) ^a^	$\Delta V_{\phi }^{0}$/ (cm^3^·mol^−1^) (in γ–CD) ^a^
0.00050	+23.1_0_	+10.5_3_	−0.03_0_
0.00100	−14.6_2_	−15.6_3_	−16.6_5_

^a^ $∆V_{\phi }^{0}$ = $V_{\phi }^{0}$_(in aqueous CDs solutions)_ − $V_{\phi }^{0}$_(in water)_. $V_{\phi }^{0}\;$ for TMP in water = 221.7_7_ cm^3^·mol^−1^ (Table S3).

**Table 9:** Viscosity *A* and *B* coefficient values in aqueous solutions of SMX and TMP without and with cyclodextrins.

System	*A* /(dm^−3/2^·mol^−1/2^)	*B* /(dm^3^·mol^−1^)	*R* ^2^
SMX/H_2_O	0.00_0_	1.20_0_	1.000
SMX/β–CD/H_2_O	−0.05_2_	3.81_2_	0.997
TMP/H_2_O	0.53_4_	2.00_7_	0.999
TMP/β–CD/H_2_O	0.12_6_	2.96_2_	0.996

**Table 10:** Statistical parameters of the SMX calibration curve in water.

λ/nm	*R* ^2^	ε/L·mol^−1^·cm^−1^	LOD/mmol·L^−1^	LOQ/mmol·L^−1^
264	0.9998	183.44	0.13	0.42

*R*^2^ represents the coefficient of determination; ε, the molar absorptivity coefficient; LOD, the limit of detection; and LOQ, the limit of quantification.

**Table 11:** Description of materials.

Chemical Name	Source	CAS Number	Mass Fraction Purity ^a^	Water Content (mass %) ^b^
Sulfamethoxazole (SMX)	TCI (Tokyo, Japan)	723-46-6	>0.98	
Trimethoprim (TMP)	Thermoscientific (Waltham, MA, USA)	738-70-5	>0.98	
α−CD	Sigma-Aldrich (Saint Louis, MO, USA)	10016-20-3	>0.98	14%
β−CD	Sigma-Aldrich	7585-39-9	>0.98	13%
γ−CD	Sigma-Aldrich	17465-86-0	>0.98	10%
Millipore-Q water (ρ = 1.82 × 10^5^ Ω m at 298.15 K)	-	7732-18-5		

^a^ As stated by the supplier. The mass fraction purity is on the water-free basis. ^b^ Determined by Karl-Fisher method by the supplier. Both purity and water content values were taken into account to determine the concentration of the different solutions used.

## Data Availability

The original contributions presented in this study are included in the article/Supplementary Material. Further inquiries can be directed at the corresponding author.

## Supplementary Materials

The following supporting information can be downloaded at: https://www.mdpi.com/article/10.3390/molecules30224359/s1, Table S1: Densities, *ρ*, and apparent molar volumes, *V**_ϕ_***, for SMX at different concentrations, *m*, in water at *T* = 298.15 K and at pressure *P* = 101.3 kPa; Table S2: Densities, *ρ*, and apparent molar volumes, *V**_ϕ_***, for SMX in (water + CDs) mixed solvents at *T* = 298.15 K and at pressure *P* = 101.3 kPa; Table S3: Densities, *ρ*, and apparent molar volumes, *V**_ϕ_***, for TMP at different concentrations, *m*, in water at *T* = 298.15 K and at pressure *P* = 101.3 kPa; Table S4: Densities, *ρ*, and apparent molar volumes, *V**_ϕ_***, for TMP in (water + CDs) mixed solvents at *T* = 298.15 K and at pressure *P* = 101.3 kPa; Table S5: Viscosity data in aqueous solutions of SMX and TMP without and with CDs.
