# Supplementary material for: Antibiotic–Cyclodextrin Interactions: An Effective Strategy for the Encapsulation of Environmental Contaminants
Source: Molecules. 2025 Nov 11;30(22):4359. doi: 10.3390/molecules30224359 (PMC12655650; doi:10.3390/molecules30224359)
Supplement: Supplementary file 1 [file molecules-30-04359-s001.zip › molecules-3959084-supplementary.pdf]

molecules

Supplementary materials

Antibiotic–Cyclodextrin Interactions: An Effective Strategy for the Encapsulation of Environmental Contaminants

Diana M. Galindres-Jiménez [1,2], Marta F. Matias [3], Isabel Paiva [4], Sónia I. G. Fangaia [3,5], Ana C. F. Ribeiro [3,*], Artur J. M. Valente [3] and Miguel A. Esteso [1,6]

[1] Facultad de Ciencias de la Salud, Universidad Católica de Ávila, Calle Los Canteros s/n, 05005 Ávila, Spain; dianam.galindres@ucavila.es (D.M.G.-J.); mangel.esteso@ucavila.es (M.A.E.)
[2] Grupo de Investigación en Ciencias y Educación (ICE), Facultad de Ingeniería, Universidad de América, Cra 1 #20-53, Bogotá 111711, Colombia
[3] CQC-IMS, Department of Chemistry, University of Coimbra, Rua Larga, 3004-535 Coimbra, Portugal; marta01fmatias@gmail.com (M.F.M.); sfangaia@fmed.uc.pt (S.I.G.F.); avalente@ci.uc.pt (A.J.M.V.)
[4] Centre of Geography and Spatial Planning, Department of Geography and Tourism, University of Coimbra, 3004-530 Coimbra, Portugal; isabelrp@fl.uc.pt
[5] Center for Innovation and Research in Oral Sciences (CIROS), Institute of Implantology and Prosthodontics, University of Coimbra, Av. Bissaya Barreto, Blocos de Celas, 3000-075 Coimbra, Portugal
[6] Unidad Docente Química Física, Universidad de Alcalá, 28805 Alcalá de Henares, Spain
* Correspondence: anacfrib@ci.uc.pt

Table of Contents:

Table S1. Densities, ρ, and apparent molar volumes, V_ϕ, for SMX at different concentrations, m, in water at T = 298.15 K and at pressure P = 101.3 kPa.

Table S2. Densities, ρ, and apparent molar volumes, V_ϕ, for SMX in (water + CDs) mixed solvents at T = 298.15 K and at pressure P = 101.3 kPa.

Table S3. Densities, ρ, and apparent molar volumes. V_ϕ, for TMP at different concentrations, m, in water at T = 298.15 K and at pressure P = 101.3 kPa. ....

Table S4. Densities, ρ, and apparent molar volumes, V_ϕ, for TMP in (water + CDs) mixed solvents at T = 298.15 K and at pressure P = 101.3 kPa.

Table S5. Viscosity data in aqueous solutions of SMX and TMP without and with cyclodextrins.

Table S1. Densities, ρ, and apparent molar volumes, V_ϕ, for SMX at different concentrations, m, in water at T = 298.15 K and at pressure P = 101.3 kPa.

$m_{(SMX)}$ / (mol·kg^{-1})	ρ [a] / (g·cm^{-3})	V_ϕ / (cm^3·mol^{-1})
0.00025	0.997064	190.1$_5$
0.00050	0.997081	188.1$_3$
0.00075	0.997100	186.1$_2$
0.00100	0.997120	184.1$_0$
		(V_ϕ^0 = 197.1$_9$) [b]

[a)] ρ^0_{H2O} (298.15 K) = 0.997048 g·cm^{-3} [26] [b)] Limiting V_ϕ value calculated by extrapolating experimental data to $m \rightarrow 0$. Standard uncertainties are: $u_r(m)$ = 1.0 ×10^{-3} (max); $u(\rho)$ = 0.150 kg·m^{-3}; $u(V_\phi)$ = 0.1 cm^3·mol^{-1}; $u(T)$ = 0.01 K; $u(P)$ = 2.03 kPa.

Table S2. Densities, *ρ*, and apparent molar volumes, V_ϕ, for SMX in (water + CDs) mixed solvents at T = 298.15 K and at pressure P = 101.3 kPa.

$m_{(\alpha\text{-CD})}$/(mol·kg^{-1})	$m_{(SMX)}$/(mol·kg^{-1})	ρ/(g·cm^{-3})	V_ϕ/(cm^3·mol^{-1})
0.00050	0.00000	0.997211	
	0.00025	0.997232	174.0_4
	0.00050	0.997252	176.0_4
	0.00075	0.997272	176.7_1
	0.00100	0.997291	178.0_5
			$V_\phi{}^0$ = 173.0_4
0.00100	0.00000	0.997382	
	0.00025	0.997402	178.0_4
	0.00050	0.997421	180.0_5
	0.00075	0.997438	183.4_0
	0.00100	0.997453	187.0_8
			$V_\phi{}^0$ = 174.5_2
**$m_{(\beta\text{-CD})}$/(mol·kg^{-1})**	**$m_{(SMX)}$/(mol·kg^{-1})**	**ρ/(g·cm^{-3})**	**V_ϕ/(cm^3·mol^{-1})**
0.00050	0.00000	0.997238	
	0.00025	0.997259	174.0_3
	0.00050	0.997279	176.0_4
	0.00075	0.997298	178.0_5
	0.00100	0.997316	180.0_6
			$V_\phi{}^0$ = 172.0_2
0.00100	0.00000	0.997439	
	0.00025	0.997458	182.0_6
	0.00050	0.997476	184.0_6
	0.00075	0.997493	186.0_7
	0.00100	0.997509	188.0_7
			$V_\phi{}^0$ = 180.0_5
**$m_{(\gamma\text{-CD})}$/(mol·kg^{-1})**	**$m_{(SMX)}$/(mol·kg^{-1})**	**ρ/(g·cm^{-3})**	**V_ϕ/(cm^3·mol^{-1})**
0.00050	0.00000	0.997274	
	0.00025	0.997294	178.0_5
	0.00050	0.997313	180.0_6
	0.00075	0.997332	180.7_3
	0.00100	0.997350	182.0_6
			$V_\phi{}^0$ = 177.0_5
0.00100	0.00000	0.997505	
	0.00025	0.997528	165.97
	0.00050	0.997549	169.99
	0.00075	0.997568	174.00
	0.00100	0.997585	178.02
			$V_\phi{}^0$ = 161.9_6

Standard uncertainties are: $u_r(m)$ = 1.0 ×10^{-3} (max); $u(\rho)$ = 0.150 kg·m^{-3}; $u(V_\phi)$ = 0.1 cm^3·mol^{-1}; $u(T)$ = 0.01 K; $u(P)$ = 2.03 kPa.

Table S3. Densities, ρ, and apparent molar volumes. V_ϕ, for TMP at different concentrations, m, in water at T = 298.15 K and at pressure P = 101.3 kPa.

$m_{(TMP)}$ /(mol·kg^{-1})	ρ [a] /(g·cm^{-3})	V_ϕ /(cm^3·mol^{-1})
0.00025	0.997065	222.7$_7$
0.00050	0.997082	224.7$_8$
0.00075	0.997098	225.4$_4$
0.00100	0.997113	226.7$_8$
		(V_ϕ^0 = 221.7$_7$) [b]

[a)] ρ^0_{H2O} (298.15 K) = 0.997048 g·cm^{-3} [26]. [b)] Limiting V_ϕ^0 values calculated by extrapolating experimental data to $m \rightarrow 0$. Standard uncertainties are: $u_r(m)$ = 1.0 ×10^{-3} (max); $u(\rho)$ = 0.150 kg·m^{-3}; $u(V_\phi)$ = 0.1 cm^3·mol^{-1}; $u(T)$ = 0.01 K; $u(P)$ = 2.03 kPa.

Table S4. Densities, ρ, and apparent molar volumes, V_ϕ, for TMP in (water + CDs) mixed solvents at T = 298.15 K and at pressure P = 101.3 kPa.

$m_{(\alpha CD)}$ /(mol·kg^{-1})	$m_{(TMP)}$/(mol·kg^{-1})	ρ/(g·cm^{-3})	V_ϕ /(cm^3·mol^{-1})
0.00050	0.00000	0.997211	
	0.00025	0.997222	246.8_8
	0.00050	0.997232	248.8_9
	0.00075	0.997241	250.9_0
	0.00100	0.997249	252.9_1
			V_ϕ^0=244.8_7
0.00100	0.00000	0.997382	
	0.00025	0.997402	210.6_6
	0.00050	0.997421	212.6_6
	0.00075	0.997438	216.0_1
	0.00100	0.997453	219.6_9
			V_ϕ^0=207.1_5
$m_{(\beta\text{-CD})}$ /(mol·kg^{-1})	$m_{(TMP)}$/(mol·kg^{-1})	ρ/(g·cm^{-3})	V_ϕ /(cm^3·mol^{-1})
0.00050	0.00000	0.997238	
	0.00025	0.997252	234.8_1
	0.00050	0.997264	238.8_3
	0.00075	0.997275	241.5_1
	0.00100	0.997285	243.8_5
			V_ϕ^0= 232.3_0
0.000100	0.00000	0.997439	
	0.00025	0.997459	210.6_5
	0.00050	0.997477	214.6_7
	0.00075	0.997493	218.6_8
	0.00100	0.997506	223.7_0
			V_ϕ^0= 206.1_4
$m_{(\gamma\text{-CD})}$/(mol kg^{-1})	$m_{(TMP)}$/(mol·kg^{-1})	ρ/(g·cm^{-3})	V_ϕ /(cm^3·mol^{-1})
0.00050	0.00000	0.997274	
	0.00025	0.997290	226.7_6
	0.00050	0.997302	232.7_9
	0.00075	0.997314	238.8_3
	0.00100	0.997322	242.8_4
			V_ϕ^0=221.7_4
0.0010	0.00025	0.997527	206.6_2
	0.00050	0.997546	208.6_3
	0.00075	0.997565	210.6_4
	0.00100	0.997584	211.6_3
			V_ϕ^0= 205.1_2

Standard uncertainties are: $u_r(m)$ = 1.0 ×10^{-3} (max); $u(\rho)$ = 0.150 kg·m^{-3}; $u(V_\phi)$ = 0.1 cm^3·mol^{-1}; $u(T)$ = 0.01 K; $u(P)$ = 2.03 kPa.

Table S5. Viscosity data in aqueous solutions of SMX and TMP without and with β-CD.

$C_{Antibiotic}$ /(mol·kg^{-1})	η_r (SMX in water)	η_r (SMX in β-CD + water)	$\Delta\eta_r$ %	η_r (TMP in water)	η_r (TMP in β-CD + water)	$\Delta\eta_r$ %
0	1.0000[a]	1.0019[b]	0.19	1.0000[a]	1.0019[b]	0.19
0.00025	1.0003	1.0020	0.17	1.0009	1.0049	0.41
0.00050	1.0006	1.0026	0.20	1.0013	1.0060	0.47
0.00075	1.0009	1.0032	0.23	1.0016	1.0076	0.60
0.00100	1.0012	1.0041	0.29	1.0019	1.0090	0.71

[a]) The relative viscosity of aqueous solutions of SMX (or TMP) was determined using the viscosity of pure water at 298.15 K as a reference. That is, η_{H2O} = 0.8904 × 10^{-3} Pa·s [29].

[b]) For the calculation of the relative viscosity in aqueous β-CD solutions of SMX (or TMP), the absolute viscosity of the 1 mM aqueous β-CD solution at 298.15 K was used as the reference. (i.e., η = 0.8921 × 10^{-3} Pa·s) [27].
